# The caspase-8/RIPK3 signaling axis in antigen presenting cells controls the inflammatory arthritic response

**DOI:** 10.1186/s13075-017-1436-4

**Published:** 2017-10-04

**Authors:** Salina Dominguez, Anna B. Montgomery, G. Kenneth Haines, Christina L. Bloomfield, Carla M. Cuda

**Affiliations:** 1 Department of Medicine, Division of Rheumatology, Northwestern University, Feinberg School of Medicine, 240 East Huron Street, M300, Chicago, IL 60611 USA; 2 Department of Pathology, Icahn School of Medicine at Mount Sinai, New York, NY 10029 USA

**Keywords:** Caspase-8, RIPK3, Rheumatoid arthritis, Dendritic cells, Macrophages

## Abstract

**Background:**

Caspase-8 is a well-established initiator of apoptosis and suppressor of necroptosis, but maintains functions beyond cell death that involve suppression of receptor-interacting serine-threonine kinases (RIPKs). A genome-wide association study meta-analysis revealed an SNP associated with risk of rheumatoid arthritis (RA) development within the locus containing the gene encoding for caspase-8. Innate immune cells, like macrophages and dendritic cells, are gaining momentum as facilitators of autoimmune disease pathogenesis, and, in particular, RA. Therefore, we examined the involvement of caspase-8 within these antigen-presenting cell populations in the pathogenesis of an arthritis model that resembles the RA effector phase.

**Methods:**

*Cre* ^LysM^ *Casp8* ^flox/flox^ and *Cre* ^CD11c^ *Casp8* ^flox/flox^ mice were bred via a cross between *Casp8* ^flox/flox^ and *Cre* ^LysM^ or *Cre* ^CD11c^ mice. *RIPK3* ^–/–^ *Cre* ^LysM^ *Casp8* ^flox/flox^ and *RIPK3* ^–/–^ *Cre* ^CD11c^ *Casp8* ^flox/flox^ mice were generated to assess RIPK3 contribution. Mice were subjected to K/BxN serum-transfer-induced arthritis. Luminex-based assays were used to measure cytokines/chemokines. Histological analyses were utilized to examine joint damage. Mixed bone marrow chimeras were generated to assess synovial cell survival. Flow cytometric analysis was employed to characterize cellular distribution. For arthritis, differences between the groups were assessed using two-way analysis of variance (ANOVA) for repeated measurements. All other data were compared by the Mann-Whitney test.

**Results:**

We show that intact caspase-8 signaling maintains opposing roles in lysozyme-M- and CD11c-expressing cells in the joint; namely, caspase-8 is crucial in CD11c-expressing cells to delay arthritis induction, while caspase-8 in lysozyme M-expressing cells hinders arthritis resolution. Caspase-8 is also implicated in the maintenance of synovial tissue-resident macrophages that can limit arthritis. Global loss of RIPK3 in both caspase-8 deletion constructs causes the response to arthritis to revert back to control levels via a mechanism potentially independent of cell death. Mixed bone marrow chimeric mice demonstrate that caspase-8 deficiency does not confer preferential expansion of synovial macrophage and dendritic cell populations, nor do caspase-8-deficient synovial populations succumb to RIPK3-mediated necroptotic death.

**Conclusions:**

These data demonstrate that caspase-8 functions in synovial antigen-presenting cells to regulate the response to inflammatory stimuli by controlling RIPK3 action, and this delicate balance maintains homeostasis within the joint.

**Electronic supplementary material:**

The online version of this article (doi:10.1186/s13075-017-1436-4) contains supplementary material, which is available to authorized users.

## Background

Rheumatoid arthritis (RA) is a prevalent chronic inflammatory autoimmune disease that begins with dysregulation of the immune system and culminates in progressive joint destruction leading to increased morbidity and mortality. While lymphocytes are crucial for the initiation of this disease [1], professional phagocytes of the innate immune system, including macrophages and dendritic cells (DCs), are also emerging as critical cell populations in the pathogenesis of RA. There are increased numbers of circulating monocytes in the peripheral blood of patients with RA [2], which in the joint differentiate into pro-inflammatory macrophages, leading to the elevation in macrophage numbers that is associated with articular destruction. Synovial macrophages are highly activated in RA, express elevated levels of toll-like receptors (TLR) 2, 3, 4 and 7 [3, 4] and contribute to inflammation and cartilage and bone destruction through the production of degradative enzymes and cytokines/chemokines. Approved therapeutic agents decrease inflammation, bone destruction and macrophage numbers in the synovial sublining [5–7]. Further, reduced macrophage number correlates with better outcomes in RA and is a potential biomarker for efficacious therapies [8, 9]. While synovial macrophages are critical for the pathogenesis of RA, the impact of DCs in the arthritic joint is less defined. DCs contribute to the marked increase in leukocyte infiltration into the synovial tissue in patients with RA [10], where they may contribute to the initiation of disease by producing cytokines and presenting arthritogenic antigens, which in combination activate autoreactive T cells [11, 12]. Evidence from human synovial tissue in RA and murine models of RA indicates that DCs drive the formation of ectopic lymphoid organs commonly found in synovial biopsies in RA [13]. A substantial portion of patients with RA present with a clear type I interferon signature, thereby potentially implicating plasmacytoid DCs in disease pathogenesis, as this population is a major producer of type I interferon [14, 15]. Irregular expression of Fc-γ-RII and hyperactive responses to stimulation of TLR2 and TLR4 are observed in dendritic cells from patients with RA with increased disease activity [16, 17]. Lower levels of circulating DCs in patients with RA suggest that plasmacytoid and myeloid DCs may selectively home to the inflamed joint [18–22]. Moreover, this decrease correlates with the presence of a population of DCs enriched for a high T cell stimulatory capacity in the inflamed synovium [18–22]. Although macrophages and DCs are clearly implicated in the pathogenesis of RA, relatively little is known about the mechanisms behind their involvement.

Caspase-8 is a cysteine-aspartic acid protease critically involved in two essential death pathways, apoptosis and necroptosis, responsible for the fate of a cell. Stimulation of a death receptor (Fas or tumor necrosis factor receptor 1 (TNFR1)) by its ligand recruits Fas-associated death domain protein (FADD) [23]. This protein aggregation then recruits pro-caspase-8, which upon dimerization becomes active. Active caspase-8 initiates the degradative phase of apoptosis through caspase-3/7 activation or blocks necroptosis via receptor-interacting serine-threonine kinase (RIPK) 1-RIPK3 suppression, depending upon the availability of cellular FADD-like interleukin (IL)-1β-converting enzyme (FLICE)-inhibitory protein (cFLIP) [23, 24]. Should levels of cFLIP be low, caspase-8 homodimers form and apoptosis ensues [24]. Conversely, high levels of cFLIP enable formation of caspase-8-cFLIP heterodimers, which limit RIPK signaling for necroptosis and prevent apoptosis [24]. In the absence of caspase-8, apoptosis cannot occur but RIPK signaling proceeds unchecked, leading to necroptosis [24]. While *RIPK1* ^-/-^ mice die perinatally [25] and *RIPK3* ^-/-^ mice show no gross defect in development [26], global deletion of either RIPK rescues the embryonic lethality associated with global knockout of caspase-8 [24, 27].

Aside from its prescribed role in necroptosis, RIPK signaling has also been implicated in cell-death-independent activities in innate immune cells. Examination of a kinase-dead mutant of RIPK1 (D138N) in macrophages suggests that RIPK1 kinase activity promotes acute inflammatory responses to lipopolysaccharide (LPS) [28]. Evaluation of an alternate kinase-dead mutant of RIPK1 (K45A) in macrophages shows that RIPK1 kinase activity plays a critical role in promoting host responses to inflammatory stimuli and cytokine signaling [29]. It has also been shown that *RIPK3* ^-/-^ DCs are highly defective in LPS-induced expression of inflammatory cytokines and contribute to a reduced response to injury-induced tissue repair in a colitis model [30]. Similar to RIPK, mounting evidence implicates caspase-8 in death-independent activities [31–36] that may also require RIPK. Our own studies supplement death-independent functions for caspase-8. Studies of *Cre* ^LysM^ *Casp8* ^flox/flox^ mice, where caspase-8 is deleted in lysozyme M-expressing cells, reveal that caspase-8 associates with RIPK1 and RIPK3 to limit its signaling following TLR activation by gut microflora and prevents the continued activation of these populations to keep systemic inflammation in check [37]. Further, *Cre* ^CD11c^ *Casp8* ^flox/flox^ mice, where caspase-8 is deleted in CD11c-expressing cells, develop systemic autoimmunity independent of DC lifespan, indicating that caspase-8 signaling in CD11c-expressing populations maintains tolerance [38]. Although RIPK3 is dispensable for this process in CD11c-expressing cells, uncontrolled TLR activation in an RIPK1-dependent manner is responsible for the enhanced functionality of caspase-8-deficient DCs [38]. Collectively, these data connect caspase-8 and RIPK not only to death, but also to death-independent inflammatory processes.

A genome-wide association study meta-analysis in more than 100,000 subjects of European and Asian ancestry (29,880 were diagnosed with RA), evaluated nearly 10 million single-nucleotide polymorphisms (SNPs) and identified a SNP associated with risk of RA development within the locus containing the gene encoding for both caspase-8 and cFLIP [39]. Despite this newly discovered link between caspase-8 and RA, the cellular mechanisms by which caspase-8 mediates this predisposition to RA are unknown. Here, we investigated how caspase-8 signaling impacts development and progression of the acute K/BxN serum-transfer-induced arthritis model of inflammatory arthritis that resembles the effector stage of RA. The K/BxN serum-transfer-induced arthritis model entails initiation, developmental/propagation and resolution phases and is advantageous because it is not T and B cell dependent but rather depends on innate immune cells, including macrophages and DCs, to mediate disease [40–44]. We showed that *Cre* ^LysM^ *Casp8* ^flox/flox^ mice resolve K/BxN serum-transfer-induced arthritis more rapidly than control mice, which suggests that caspase-8 in this context prolongs the inflammatory response. In stark contrast to *Cre* ^LysM^ *Casp8* ^flox/flox^ mice, *Cre* ^CD11c^ *Casp8* ^flox/flox^ mice exhibited a more rapid and severe onset of arthritis, indicating that in this caspase-8-deletion construct, caspase-8 controls the magnitude of the initial inflammatory response. Further, in *Cre* ^CD11c^ *Casp8* ^flox/flox^ mice, caspase-8 is implicated in the maintenance of synovial tissue-resident macrophages that can limit arthritis development. We observed that global deletion of RIPK3 in both of our caspase-8 deletion constructs (*Cre* ^LysM^ *Casp8* ^flox/flox^ and *Cre* ^CD11c^ *Casp8* ^flox/flox^) caused the response to K/BxN serum-transfer-induced arthritis to revert back to that of control *Casp8* ^flox/flox^ mice, potentially independent of controlling necroptosis. These data suggest that deletion of caspase-8 leads to unchecked action of RIPK3, and this delicate balance maintains homeostasis within the joint.

## Methods

### Mice

Male KRN mice were kindly provided by Dr. Diane Mathis and were crossed with female NOD mice (Taconic) for the generation of K/BxN serum. All subsequently described strains are on a C57BL/6 (B6) background. Mice homozygous for the loxP flanked Caspase-8 allele (*Casp8* ^flox/flox^; generated on a 129 background and backcrossed to B6 for at least 12 generations [45, 46]) were crossed with mice expressing Cre under control of either the murine lysozyme M gene promoter (*Cre* ^LysM^; generated on a 129 background, backcrossed to B6 for at least six generations and homozygous for Cre, Jackson Laboratory 004781) or CD11c promoter (*Cre* ^CD11c^; generated on a B6 background and transgene positive for Cre, Jackson Laboratory 007567, Alexander Chervonsky) to generate *Cre* ^LysM^ *Casp8* ^flox/flox^ (homozygous for Cre, homozygous for floxed caspase-8) and *Cre* ^CD11c^ *Casp8* ^flox/flox^ (transgene positive for Cre, homozygous for floxed caspase-8) mice, as previously described [38, 47]. *Cre* ^LysM^ *Casp8* ^flox/flox^ and *Cre* ^CD11c^ *Casp8* ^flox/flox^ mice were crossed with *RIPK3* ^–/–^ (homozygous *RIPK3* ^–/–^, generated on a 129 background and backcrossed to B6 for at least four generations, Genentech [26]) to generate *RIPK3* ^–/–^ *Cre* ^LysM^ *Casp8* ^flox/flox^ (homozygous *RIPK3* ^–/–^, homozygous for Cre, homozygous for floxed caspase-8) and *RIPK3* ^–/–^ *Cre* ^CD11c^ *Casp8* ^flox/flox^ (homozygous *RIPK3* ^–/–^, transgene positive for Cre, homozygous for floxed caspase-8) mice as previously described [38, 47]. *RIPK3* ^–/–^ *Casp8* ^flox/flox^ (homozygous *RIPK3* ^–/–^, homozygous for floxed caspase-8) mice were also generated as a control. B6.*CD45.1*/*2* mice were generated from a cross of B6 (Jackson Laboratory) and B6.*CD45*.1 mice. Mice were housed at a barrier-free and specific pathogen-free facility at the Center for Comparative Medicine at Northwestern University (Chicago, IL, USA). All lines were bred homozygously. Weanlings homozygous for *RIPK3* ^–/–^, *Casp8* ^flox/flox^, and/or *Cre* ^LysM^ constructs or transgene positive for the *Cre* ^CD11c^ construct where applicable were kept for experiments. Male mice were used in all studies and housed according to strain. Transnetyx (Memphis, TN, USA) performed all genotyping of mice and analysis of caspase-8 gene deletion in sorted cell populations. All procedures were approved by the Institutional Animal Care and Use committee at Northwestern University.

### K/BxN serum-transfer-induced arthritis

K/BxN serum-transfer-induced arthritis was induced by intravenous injection of 75 μL of arthritogenic serum from 8-week-old progeny of KRN and NOD mice (K/BxN) mice [48]. K/BxN mice (transgene positive offspring of KRN and NOD mice) develop severe, spontaneous, symmetric, erosive and chronic arthritis that mimics RA [49], and disease may be passively transferred via a single intravenous injection of serum obtained from these mice. Change in ankle width was monitored using a caliper (Fowler Tools of Canada). In addition, a clinical score was determined from the sum of clinical scores for each paw (0 – no arthritis, 1 – mild arthritis, foot maintains a V-shape, 2 – moderate arthritis, foot no longer maintains a V-shape, 3 – severe arthritis). Each experiment was performed two to three times to confirm reproducibility. Whenever possible, scoring was performed in a blinded manner. At days 3, 7, 11 or 14 days post injection, mice were killed, serum was collected via cardiac puncture and ankles were harvested for fixation in 10% formalin or flow cytometric analysis.

### Mixed bone marrow chimeras

Bone marrow was aseptically harvested from tibias, femurs and humeri from 9-week-old mice, erythrocytes lysed (BD Pharm Lyse buffer) and cells incubated with Fc-block followed by incubation with fluorochrome-conjugated antibodies against B220, CD4, CD8, CD11b, Ly6G, NK1.1, Siglec F, Ter119, c-Kit and Sca-1 (BD Biosciences, eBioscience, Biolegend), as previously described [38]. Cell suspensions were sorted by fluorescence-activated cell sorting (FACS) to obtain the Lin^-^Sca-1^+^c-kit ^+^ (LSK) population. Two-month-old B6.*CD45.1* mice received a single 1000-cGy γ-irradiation dose using a Cs-137-based Gammacell-40 irradiator (Nordion). After 12 hours, 5 × 10^4^ LSK cells were intravenously injected from: *Casp8* ^flox/flox^, *Cre* ^CD11c^ *Casp8* ^flox/flox^, *RIPK3* ^–/–^ *Cre* ^CD11c^ *Casp8* ^flox/flox^, a mixture of *Casp8* ^flox/flox^ plus B6.*CD45.1*/*2*, *Cre* ^CD11c^ *Casp8* ^flox/flox^ plus B6.*CD45.1*/*2* or *RIPK3* ^–/–^ *Cre* ^CD11c^ *Casp8* ^flox/flox^ plus B6.*CD45.1*/*2* (1:1 ratio). Chimeric mice were maintained on Trimetoprim/Sulfamethoxazole (40 mg/5 mg, respectively; Hi-Tech Pharmacal) diluted in autoclaved water (2 mL antibiotics/500 mL water) and phenotyped 2 months post transfer.

### Flow cytometry

Blood was collected into EDTA-containing tubes via cardiac puncture from euthanized animals. Whole blood was stained with fluorochrome-conjugated antibodies and erythrocytes were then lysed using BD FACS lysing solution (BD Biosciences). Flow cytometric analysis of the ankles was performed as previously described [50]. Briefly, ankles were cut 3 mm above the heel and skin was removed from the feet. To avoid contamination with bone marrow cells, the bone marrow cavity in the tibia was thoroughly flushed with Hanks balanced salt solution (HBSS), the finger joints were disarticulated by pulling with blunt forceps and the tibiotalar joint was opened via the posterior access route to expose the synovial lining. The feet were incubated in digestion buffer (2.4 mg/mL dispase II, 2 mg/mL collagenase D and 0.2 mg/mL of DNase I in HBSS) for 60 min at 37 °C. Cells released during the digestion were filtered through a 40-μm nylon mesh, erythrocytes were lysed using BD Pharm Lyse (BD Biosciences) and cells were counted using the Countess automated cell counter (Invitrogen); dead cells were discriminated using trypan blue. Cells were stained with the eFluor 506 viability dye (eBioscience), incubated with FcBlock (BD Bioscience) and stained with fluorochrome-conjugated antibodies (see Additional file 1: Table S1 for the list of antibodies, clones, fluorochromes and manufacturers). Data from blood and ankles were acquired on BD LSR II flow cytometer (BD Biosciences, San Jose, CA, USA), and at least 200,000 events were acquired. Compensation and analysis of the flow cytometric data were performed using FlowJo software (TreeStar, Ashland, OR, USA). “Fluorescence minus one” controls were used when necessary to set up gates. Expression of the activation markers was presented as median fluorescence intensity (MFI). To assess deletion of caspase-8 in synovial antigen-presenting cell populations, CD11b^+^ DCs, major histocompatibility complex (MHC) II^+^ macrophages and MHC II^–^ macrophages were sorted (average purity of 97%) at the Northwestern University RLHCCC Flow Cytometry Core Facility on a BD FACSAria III instrument (BD Biosciences, San Jose, CA, USA) and analyzed for the presence of the *Casp8* ^floxed^ allele by Transnetyx.

### Cytokine measurements and histologic analysis

Serum was screened for cytokines/chemokines using a mouse Procarta Cytokine Assay Kit (Affymetrix) according to the manufacturer’s instructions. The fixed ankles were decalcified in ethylenediaminetetraacetic acid (Sigma-Aldrich, St. Louis, MO, USA). The ankles were embedded in paraffin, sectioned and 5-μm sections were stained with hematoxylin and eosin (H&E) at the Northwestern University Mouse Histology and Phenotyping Laboratory. Histopathologic scoring was performed as described [51] by a pathologist (GKH) blinded to the study, using an Olympus BS40 microscope (Olympus, Tokyo, Japan). Images were taken on an Olympus BX41 microscope equipped with a DP20 Digital Camera (Olympus) at magnification × 40 or × 100.

### Statistical analysis

Statistical analyses were performed using the GraphPad Prism 7.0 Software. Data are represented as mean ± SEM. For arthritis experiments, differences in ankle width and clinical score between the groups were compared using two-way analysis of variance (ANOVA) for repeated measurements with the Bonferroni post-hoc test. For all other analyses, differences between groups were assessed using the Mann-Whitney test.

## Results

### *Cre*^LysM^*Casp8*^flox/flox^ mice display accelerated resolution, while *Cre*^CD11c^*Casp8*^flox/flox^ mice exhibit accelerated initiation, of K/BxN serum-transfer-induced arthritis

**Fig. 1:**
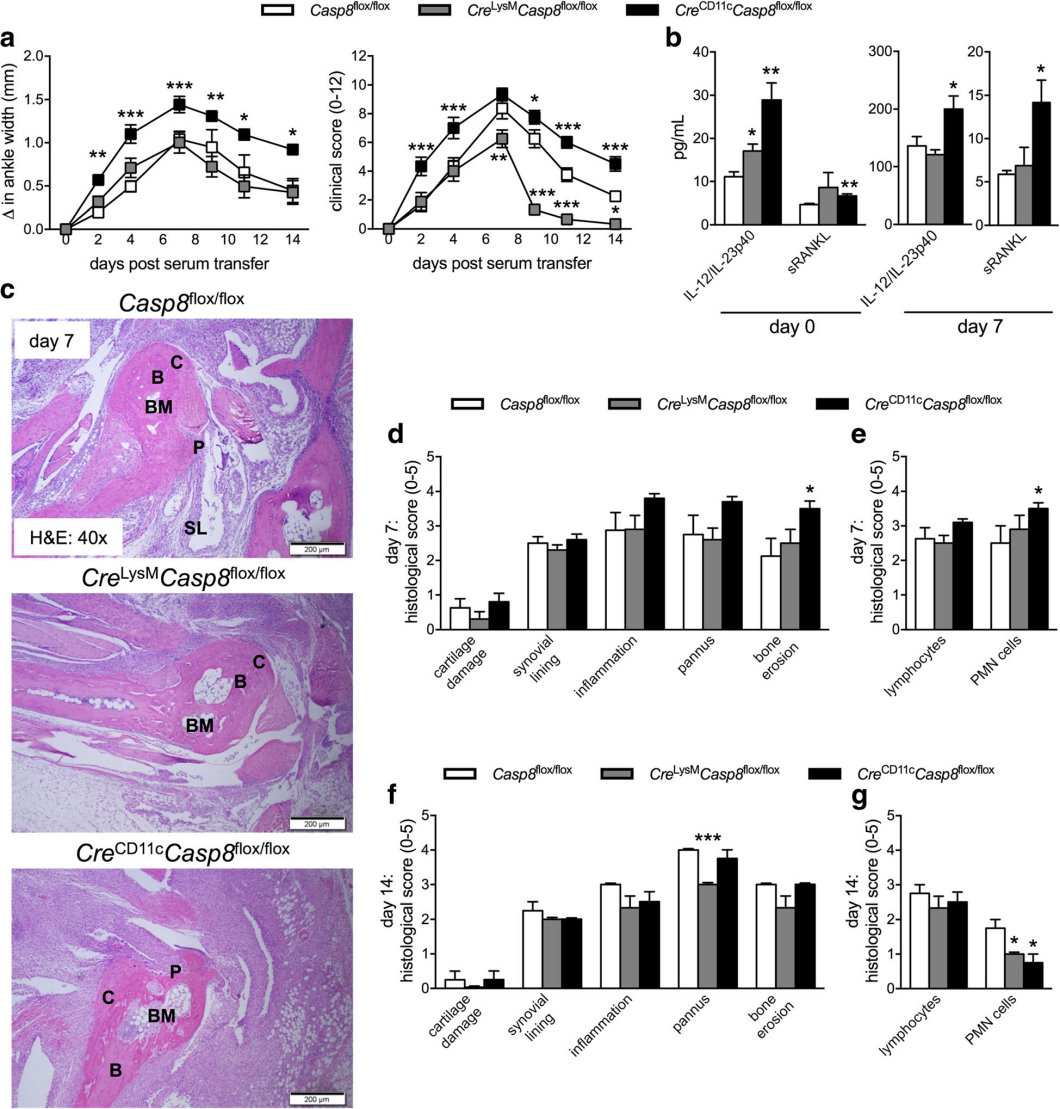
*Cre* ^LysM^ *Casp8* ^flox/flox^ mice display accelerated resolution and *Cre* ^CD11c^ *Casp8* ^flox/flox^ mice exhibit accelerated initiation of K/BxN serum-transfer-induced arthritis. Male *Casp8* ^flox/flox^ (control, n = 15), *Cre* ^LysM^ *Casp8* ^flox/flox^ (n = 16) and *Cre* ^CD11c^ *Casp8* ^flox/flox^ (n = 16) mice, 10 − 12 weeks old, were intravenously injected with K/BxN serum. **a** Depicted are combined “change in ankle width” and “clinical score” from two individual experiments. Differences between control and *Cre* ^LysM^ *Casp8* ^flox/flox^ or *Cre* ^CD11c^ *Casp8* ^flox/flox^ mice are compared by two-way analysis of variance with the Bonferroni post-hoc test: *p < 0.05; **p < 0.005; ***p < 0.0005. **b** Day-0 and day-7 serum cytokine levels from control (n = 5), *Cre* ^LysM^ *Casp8* ^flox/flox^ (n = 5) and *Cre* ^CD11c^ *Casp8* ^flox/flox^ (n = 5) mice. **c** Day-7 ankles stained with hematoxylin and eosin (H&E). Day 7P = pannus; SL = synovial lining; C = cartilage; B = bone; BM = bone marrow. Histologic scoring of H&E-stained ankle sections from day-7 control (n = 8), *Cre* ^LysM^ *Casp8* ^flox/flox^ (n = 10) and *Cre* ^CD11c^ *Casp8* ^flox/flox^ (n = 10) (**d**, **e**) and day-14 control (n = 4), *Cre* ^LysM^ *Casp8* ^flox/flox^ (n = 4) and *Cre* ^CD11c^ *Casp8* ^flox/flox^ (n = 4) (**f**, **g**). Data are means ± SEM and differences between control and *Cre* ^LysM^ *Casp8* ^flox/flox^ or *Cre* ^CD11c^ *Casp8* ^flox/flox^ mice were compared by the Mann-Whitney test: *p < 0.05; ***p < 0.0005. Casp8 caspase-8, IL, interleukin, PMN polymorphonuclear cells

Since we have previously published that caspase-8 signaling in myeloid cells and DCs is critical to suppress systemic inflammation and caspase-8 has been linked to RA susceptibility, the role that cell-specific caspase-8 plays in the development of acute inflammation in young mice prior to overt development of autoimmune disease was evaluated using the K/BxN serum-transfer-induced arthritis model. *Cre* ^LysM^ *Casp8* ^flox/flox^ mice presented with an accelerated resolution and reduced severity of K/BxN serum-transfer-induced arthritis compared to *Casp8* ^flox/flox^ mice beginning at day 7 following injection (Fig. 1a). In contrast, *Cre* ^CD11c^ *Casp8* ^flox/flox^ mice had enhanced initiation and exacerbated severity of K/BxN serum-transfer-induced arthritis compared to *Casp8* ^flox/flox^ mice (Fig. 1a), which was most pronounced at days 2 and 4 following injection. Circulating cytokine levels were assessed because elevated cytokine production may contribute to the inflammation and destruction within the joint (Fig. 1b and Additional file 1: Figure S1). Heightened IL-12/IL-23p40 and serum RANKL (sRANKL) levels were found in *Cre* ^LysM^ *Casp8* ^flox/flox^ and *Cre* ^CD11c^ *Casp8* ^flox/flox^ serum compared to control serum, which only persists in *Cre* ^CD11c^ *Casp8* ^flox/flox^ mice till day 7 (Fig. 1b). Evidence of increased bone erosion was detected in *Cre* ^CD11c^ *Casp8* ^flox/flox^ ankles harvested at 7 and 14 days following injection of K/BxN serum (Fig. 1c-d). Further, higher levels of polymorphonuclear (PMN) cells were observed in the joints of *Cre* ^CD11c^ *Casp8* ^flox/flox^ mice at day 7 (Fig. 1e). Examination of structural damage during the resolution phase of arthritis at day 14 showed that while *Cre* ^CD11c^ *Casp8* ^flox/flox^ ankles mirror *Casp8* ^flox/flox^ ankles, pannus formation subsided more quickly in *Cre* ^LysM^ *Casp8* ^flox/flox^ joints, which was consistent with accelerated disease resolution (Fig. 1c, f). Further, fewer PMN cells were identified in *Cre* ^CD11c^ *Casp8* ^flox/flox^ and *Cre* ^LysM^ *Casp8* ^flox/flox^ joints compared to *Casp8* ^flox/flox^ joints by day 14 (Fig. 1g). Taken together, these data suggest that caspase-8 deficiency can either accelerate the resolution of, or enhance the initial magnitude of, acute inflammatory arthritis depending on the deletion construct.

### Caspase-8 deficiency alters circulating and synovial populations of *Cre*^LysM^*Casp8*^flox/flox^ and *Cre*^CD11c^*Casp8*^flox/flox^ mice at steady state

**Fig. 2:**
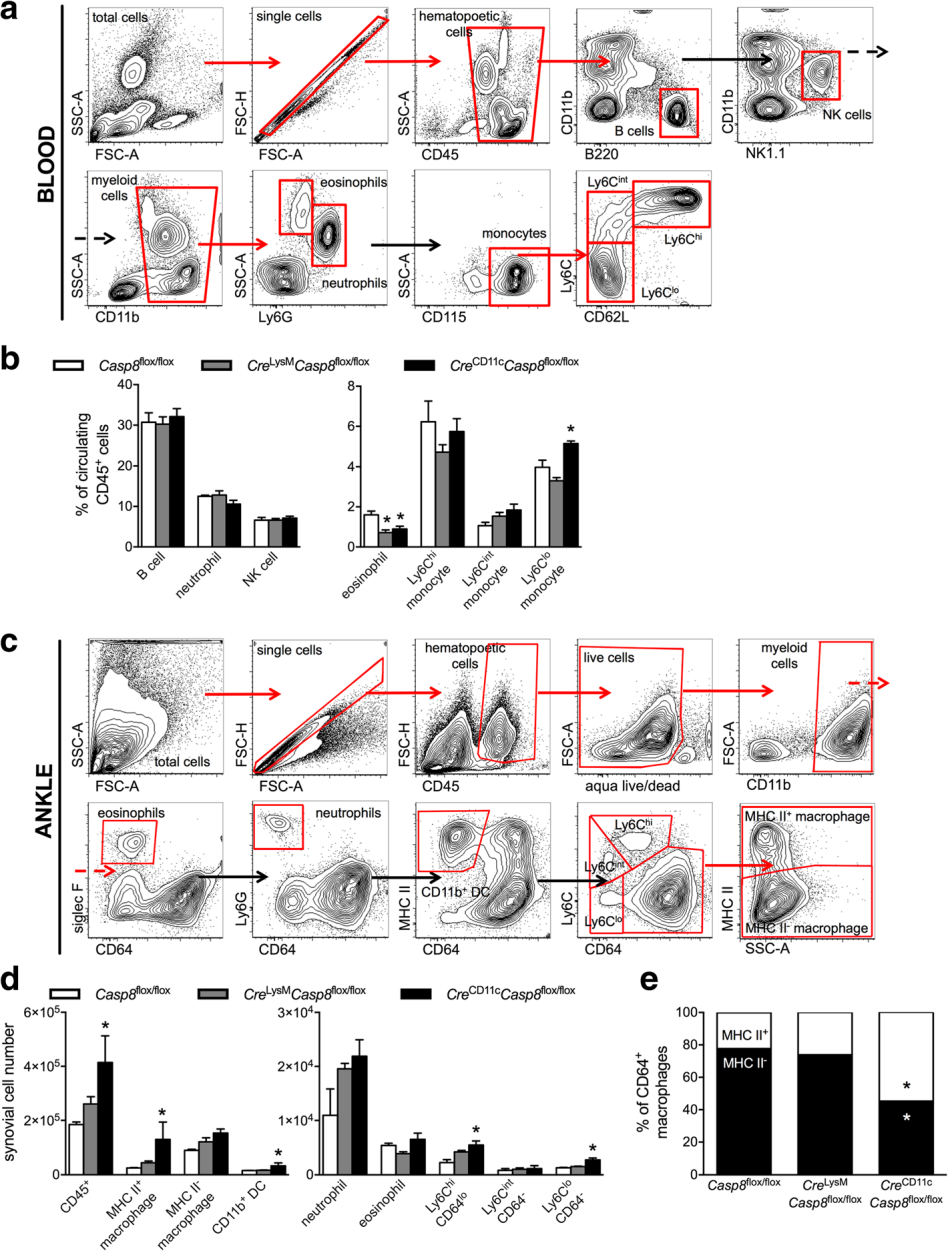
Caspase-8 deficiency alters synovial populations in the circulation and the joints at steady state. **a**, **b** Blood from naïve 10–12-week-old male *Casp8* ^flox/flox^ (control, n = 4), *Cre* ^LysM^ *Casp8* ^flox/flox^ (n = 5) and *Cre* ^CD11c^ *Casp8* ^flox/flox^ (n = 5) mice were analyzed by flow cytometric analysis. **a** Fluorescence-activated cell sorting (FACS) plots from the naïve circulation. Red arrows denote the sequential gated population (red boxes). Black arrows denote he sequential non-gated population. **b** Cellular distribution represented as the percentage of circulating CD45^+^ cells. **c**, **d** Ankles from naïve 10–12-week-old male *Casp8* ^flox/flox^ (control, n = 3), *Cre* ^LysM^ *Casp8* ^flox/flox^ (n = 3) and *Cre* ^CD11c^ *Casp8* ^flox/flox^ (n = 4) mice were analyzed by flow cytometric analysis. **c** FACS plots from a naïve joint. Red arrows denote the sequential gated population (red boxes). Black arrows denote the sequential non-gated population. **d** Synovial cell distribution presented as numbers of cells. **e** Proportion of macrophages (CD11b^+^CD64^+^) that are major histocompatibility complex (MHC) II^+^ and MHC II^–^. Data are means ± SEM, representative of two individual studies and differences between control and *Cre* ^LysM^ *Casp8* ^flox/flox^ or *Cre* ^CD11c^ *Casp8* ^flox/flox^ mice were compared by the Mann-Whitney test: *p < 0.05; **p < 0.005. Casp8 caspase-8, NK natural killer, DC dendritic cells

We have previously shown that the pathogenesis of the K/BxN serum-transfer-induced model of arthritis depends upon the Ly6C^lo^ monocyte population, which patrol the luminal side of the endothelium and extravasate into the joint during the initiation phase of arthritis preceding the massive neutrophil influx [50]. These recruited cells not only differentiate into classically activated macrophages to drive joint destruction, but also possess the capacity to switch after differentiation to an alternatively activated phenotype to assist in the resolution of inflammation [50]. Further, there are long-lived radio-resistant tissue-resident MHC II^–^ macrophages in the naïve joint, which do not require input from the bone marrow to maintain their population [50]. During K/BxN serum-transfer-induced arthritis, these tissue-resident MHC II^–^ macrophages can limit the initial severity of arthritis and play a role in maintaining joint integrity [50]. Our data suggest that a differential response to inflammatory arthritis occurs depending on the caspase-8 deletion construct. We therefore examined whether a preexisting disruption of the cellular composition of the circulation and/or the synovium may potentially account for this variable outcome following induction of K/BxN serum-transfer-induced arthritis. B cell, neutrophil and natural killer (NK) cell population levels were consistent between all strains, but circulating eosinophils were decreased in the circulation of naïve *Cre* ^LysM^ *Casp8* ^flox/flox^ and *Cre* ^CD11c^ *Casp8* ^flox/flox^ mice compared to *Casp8* ^flox/flox^ mice using 10-color flow cytometric analysis (Fig. 2a, b). Since circulating monocytes extravasate into tissues following an inflammatory insult and differentiate into tissue macrophages, we also examined levels of the monocyte subsets in control and experimental strains. While Ly6C^hi^ and Ly6C^int^ monocyte levels of naïve *Cre* ^LysM^ *Casp8* ^flox/flox^ and *Cre* ^CD11c^ *Casp8* ^flox/flox^ mice resembled those of control *Casp8* ^flox/flox^ mice, *Cre* ^CD11c^ *Casp8* ^flox/flox^ mice had increased levels of Ly6C^lo^ monocytes (Fig. 2b). These data suggest that deletion of caspase-8 in CD11c-expressing cells leads to increased levels of Ly6C^lo^ monocytes that are crucial to instigating the initial inflammation observed following induction of K/BxN serum-transfer-induced arthritis.

We then determined the cellular composition of naïve synovium of *Casp8* ^flox/flox^, *Cre* ^LysM^ *Casp8* ^flox/flox^ and *Cre* ^CD11c^ *Casp8* ^flox/flox^ mice using 10-color flow cytometric analysis (Fig. 2c-e). While the cellular distribution of the naïve synovium of *Cre* ^LysM^ *Casp8* ^flox/flox^ mice mimicked that of control *Casp8* ^flox/flox^ mice, *Cre* ^CD11c^ *Casp8* ^flox/flox^ mice had increased CD45^+^ hematopoietic cells in the joint at steady state (Fig. 2d). Examination of this increased hematopoietic population revealed that the synovium of *Cre* ^CD11c^ *Casp8* ^flox/flox^ mice contained elevated numbers of MHC II^+^ macrophages and CD11b^+^ DCs. Further, increased numbers of Ly6C^hi^CD64^lo^ and Ly6C^lo^CD64^-^ cells were also observed in *Cre* ^CD11c^ *Casp8* ^flox/flox^ joints compared to control *Casp8* ^flox/flox^ joints (Fig. 2d). While the macrophage distribution from *Cre* ^LysM^ *Casp8* ^flox/flox^ and *Casp8* ^flox/flox^ joints were indistinguishable, *Cre* ^CD11c^ *Casp8* ^flox/flox^ joints exhibited an increased proportion of MHC II^+^ macrophages and a correspondingly reduced proportion of MHC II^–^ tissue-resident macrophages potentially capable of limiting arthritis progression. Taken together, these data implicate caspase-8 in CD11c-expressing cells in the maintenance of pathogenic Ly6C^lo^ monocytes and anti-inflammatory synovial tissue-resident macrophages.

### RIPK3 signaling contributes to the dysregulated responses to K/BxN serum-transfer-induced arthritis in *Cre*^LysM^*Casp8*^flox/flox^ and *Cre*^CD11c^*Casp8*^flox/flox^ mice

**Fig. 3:**
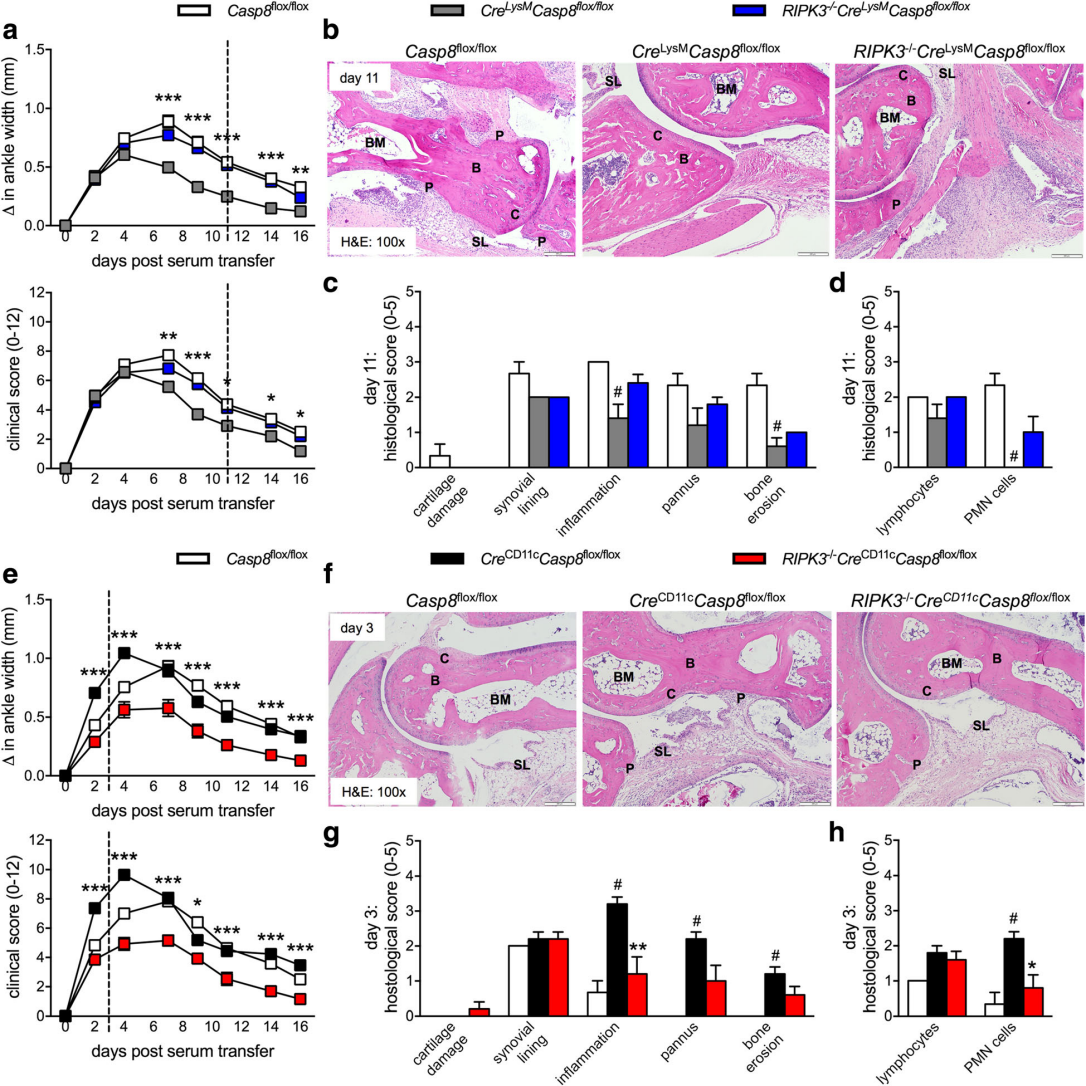
Receptor-interacting serine-threonine kinase 3 (RIPK3) signaling contributes to the aberrant response to K/BxN serum-transfer-induced arthritis in *Cre* ^LysM^ *Casp8* ^flox/flox^ and *Cre* ^CD11c^ *Casp8* ^flox/flox^ mice. **a**-**d** Male 10 − 12-week-old *Casp8* ^flox/flox^ (control, n = 17), *Cre* ^LysM^ *Casp8* ^flox/flox^ (n = 28) and *RIPK3* ^–/–^ *Cre* ^LysM^ *Casp8* ^flox/flox^ (n = 33) mice were intravenously injected with K/BxN serum. **a** Depicted are combined “change in ankle width” and “clinical score” from three individual experiments. Differences between *Cre* ^LysM^ *Casp8* ^flox/flox^ and *RIPK3* ^–/–^ *Cre* ^LysM^ *Casp8* ^flox/flox^ mice were tested by two-way analysis of variance with the Bonferroni post-hoc test: *p < 0.05; **p < 0.005; ***p < 0.0005. **b** Day-11 ankles from control (n = 3), *Cre* ^LysM^ *Casp8* ^flox/flox^ (n = 5) and *RIPK3* ^–/–^ *Cre* ^LysM^ *Casp8* ^flox/flox^ (n = 5) mice stained with hematoxylin and eosin (H&E). P = pannus; SL = synovial lining; C = cartilage; B = bone; BM = bone marrow. **c**, **d** Histologic scoring of day-11 H&E-stained ankle sections. Data are means ± SEM and are compared between control and *Cre* ^LysM^ *Casp8* ^flox/flox^ mice by the Mann-Whitney test: ^#^ *p* < 0.05. **e**-**h** Male 10–12-week-old control (n = 20), *Cre* ^CD11c^ *Casp8* ^flox/flox^ (n = 26) and *RIPK3* ^–/–^ *Cre* ^CD11c^ *Casp8* ^flox/flox^ (n = 21) mice were intravenously injected with K/BxN serum. **e** Depicted are combined “change in ankle width” and “clinical score” from three individual experiments. Differences between *Cre* ^CD11c^ *Casp8* ^flox/flox^ and *RIPK3* ^–/–^ *Cre* ^CD11c^ *Casp8* ^flox/flox^ mice were compared by two-way analysis of variance with the Bonferroni post-hoc test: *p < 0.05; ***p < 0.0005. **f** Day-3 ankles stained with H&E. **g**, **h** Histologic scoring of day-3 H&E-stained ankle sections from from control (n = 3), *Cre* ^CD11c^ *Casp8* ^flox/flox^ (n = 5) and *RIPK3* ^–/–^ *Cre* ^CD11c^ *Casp8* ^flox/flox^ (n = 5) mice. Data are means ± SEM and were compared between control and *Cre* ^CD11c^ *Casp8* ^flox/flox^ mice by the Mann-Whitney test: ^#^ *p* < 0.05 and between *Cre* ^CD11c^ *Casp8* ^flox/flox^ and *RIPK3* ^–/–^ *Cre* ^CD11c^ *Casp8* ^flox/flox^ mice by the Mann-Whitney test: **p < 0.005; ***p < 0.0005

Caspase-8 is an endogenous suppressor of RIPK signaling; therefore, we examined whether unchecked RIPK3 signaling may contribute to the aberrant responses to K/BxN serum-transfer-induced arthritis observed in our *Cre* ^LysM^ *Casp8* ^flox/flox^ and *Cre* ^CD11c^ *Casp8* ^flox/flox^ caspase-8-deficient mice. We utilized our previously published *RIPK3* ^–/–^ *Cre* ^LysM^ *Casp8* ^flox/flox^ and *RIPK3* ^–/–^ *Cre* ^CD11c^ *Casp8* ^flox/flox^ strains [37, 38]. Further, we included analysis of K/BxN serum-transfer-induced arthritis in B6, *RIPK3* ^–/–^ and *RIPK3* ^–/–^ *Casp8* ^flox/flox^ control mice (Additional file 1: Figure S2). We observed no difference between control *Casp8* ^flox/flox^ mice and B6, *RIPK3* ^–/–^ or *RIPK3* ^–/–^ *Casp8* ^flox/flox^ strains, though we detected slower induction of arthritis in *RIPK3* ^–/–^ *Casp8* ^flox/flox^ mice compared to *RIPK3* ^–/–^ mice. Global deletion of RIPK3 in *Cre* ^LysM^ *Casp8* ^flox/flox^ mice was sufficient to restore the inflammatory response to K/BxN serum-transfer-induced arthritis to that of the control *Casp8* ^flox/flox^ mice (Fig. 3a). The reduction in overall histologically identified inflammation, bone erosion and influx of PMN cells in *Cre* ^LysM^ *Casp8* ^flox/flox^ mice was reversed, though not to the level of significance (Fig. 3b-d). Deletion of RIPK3 in *Cre* ^CD11c^ *Casp8* ^flox/flox^ mice reversed the response to K/BxN serum-transfer-induced arthritis to levels below that of *Casp8* ^flox/flox^ mice (Fig. 3e). Further, joint inflammation and influx of PMN cells were prevented by deletion of RIPK3 in *Cre* ^CD11c^ *Casp8* ^flox/flox^ mice (Fig. 3f-h). These data indicate that the dysregulated response to K/BxN serum-transfer-induced arthritis observed in *Cre* ^LysM^ *Casp8* ^flox/flox^ and *Cre* ^CD11c^ *Casp8* ^flox/flox^ mice requires RIPK3 signaling.

### The caspase-8-RIPK3 signaling axis functions in the naive joint independent of cell death

Our previous publications show that in *Cre* ^LysM^ *Casp8* ^flox/flox^ splenocytes, caspase-8 deletion is restricted to CD11b^+^Ly6G^+^ neutrophils, the CD11b^+^F4/80^+^ Ly6C^hi^ and Ly6C^lo^ monocyte/macrophage populations and a portion of tissue-resident CD11b^-^F4/80^+^ red pulp macrophages, but not conventional DC subsets. In contrast, deletion of caspase-8 in *Cre* ^CD11c^ *Casp8* ^flox/flox^ splenocytes is restricted to conventional DC subsets and a portion of tissue-resident CD11b^-^F4/80^+^ red pulp macrophages, but not neutrophils or CD11b^+^F4/80^+^ Ly6C^hi^ and Ly6C^lo^ monocyte/macrophage populations [37, 38]. Previous reports suggest that cre recombinase constructs that utilize the lysozyme M and CD11c promoters are not entirely cell-specific [52]. Therefore, we examined deletion of caspase-8 in tissue-resident antigen-presenting cell populations within the joint of control and experimental strains. Caspase-8 was at least 75% deleted in the MHC II^+^ and MHC II^–^ macrophage populations and the CD11b^+^ DC population of both naïve *Cre* ^LysM^ *Casp8* ^flox/flox^ and *Cre* ^CD11c^ *Casp8* ^flox/flox^ joints (Additional file 1: Figure S3). These data suggest that lysozyme M and CD11c are turned on in both tissue-resident synovial macrophages and DCs. To eliminate the potential confounder of caspase-8-deficient neutrophils and monocytes entering the joint of *Cre* ^LysM^ *Casp8* ^flox/flox^ mice, we focused our attention on the *Cre* ^CD11c^ *Casp8* ^flox/flox^ strain.

**Fig. 4:**
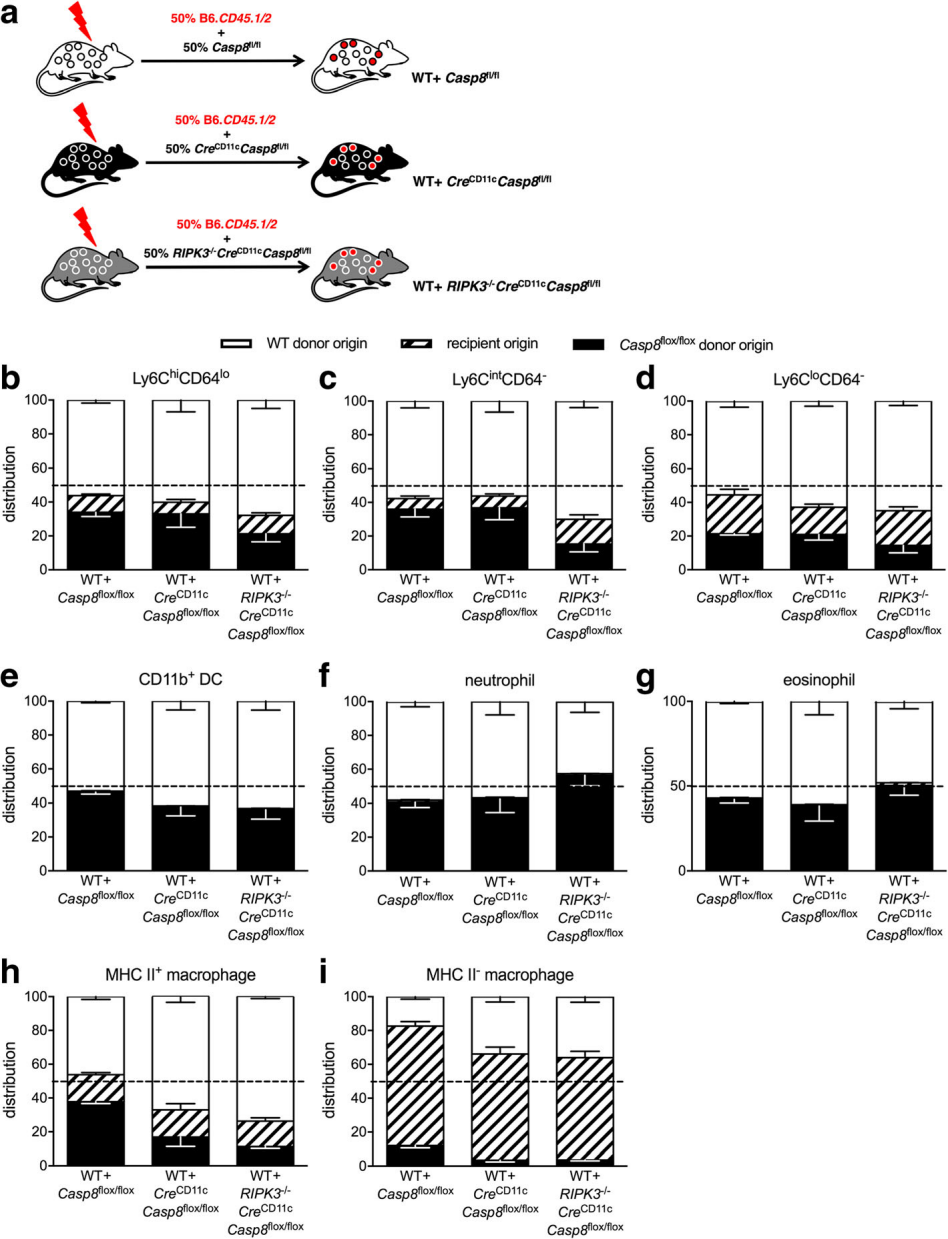
The caspase-8-receptor-interacting serine-threonine kinase 3 (RIPK3) signaling axis functions in the naive joint independent of cell death. B6.*CD45.1* mice reconstituted with equal portions of B6.*CD45.1*/*2* (wild-type (WT)) and either *Casp8* ^flox/flox^ (control, n = 5), *Cre* ^CD11c^ *Casp8* ^flox/flox^ (n = 4) or *RIPK3* ^–/–^ *Cre* ^CD11c^ *Casp8* ^flox/flox^ (n = 4) fluorescence-activated cell sorting (FACS)-sorted Lin^-^Sca-1^+^c-kit ^+^ (LSK) populations were maintained on low-dose oral antibiotics. Data are representative of two individual studies. **a** Representation of chimera generation. Chimeric mice were evaluated 8 weeks post-transfer for distribution of WT (45.1/2) and control, *Cre* ^CD11c^ *Casp8* ^flox/flox^ or *RIPK3* ^–/–^ *Cre* ^CD11c^ *Casp8* ^flox/flox^ (45.2)-derived synovial populations of Ly6C^hi^CD64^lo^ cells (**b**), Ly6C^int^CD64^-^ cells (**c**), Ly6C^lo^CD64^-^ cells (**d**), CD11b^+^ dendritic cells (**e**), neutrophils (**f**), eosinophils (**g**), MHC II^+^ macrophages (**h**) and MHC II^–^ macrophages (**i**). Error bars for 45.1/2-derived and 45.2-derived synovial populations are directed down; error bars for recipient-derived synovial populations are directed up. PMN polymorphonuclear cells

Previous studies in lymphocytes show that loss of caspase-8 results in RIPK3-mediated necroptosis [53]. Although caspase-8 is known to regulate death, evidence implicates caspase-8 in cell-specific death-independent processes that rely on RIPK suppression. Therefore, we generated mixed bone marrow chimeric mice to determine how the caspase-8-RIPK3 signaling axis affects synovial cell distribution (Fig. 4a). *Cre* ^CD11c^ *Casp8* ^flox/flox^-derived subsets of synovial Ly6C^hi^CD64^lo^, Ly6C^int^CD64^-^ and Ly6C^lo^CD64^-^ cells, CD11b^+^ DCs, neutrophils and eosinophils were found at similar proportions compared to wild-type (WT)-derived subsets in mixed chimeric mice (Additional file 1: Figure S4 and Fig. 4b-g). However, *Cre* ^CD11c^ *Casp8* ^flox/flox^-derived MHC II^+^ and MHC II^–^ macrophage subsets were observed at decreased proportions compared to WT-derived subsets in mixed chimeric mice (Additional file 1: Figure S4 and Fig. 4h-i). Deletion of RIPK3 did not reverse this abnormality, as the proportion of *RIPK3* ^–/–^ *Cre* ^CD11c^ *Casp8* ^flox/flox^-derived MHC II^+^ and MHC II^–^ macrophage subsets in mixed chimeric mice were not restored to WT proportions. Taken together, these data suggest that caspase-8 in synovial macrophage and DC populations plays only a minor role in the survival of these cells.

### Deletion of RIPK3 reverses the cellular dysregulation in the joint under steady state and arthritic conditions in *Cre*^CD11c^*Casp8*^flox/flox^ mice

**Fig. 5:**
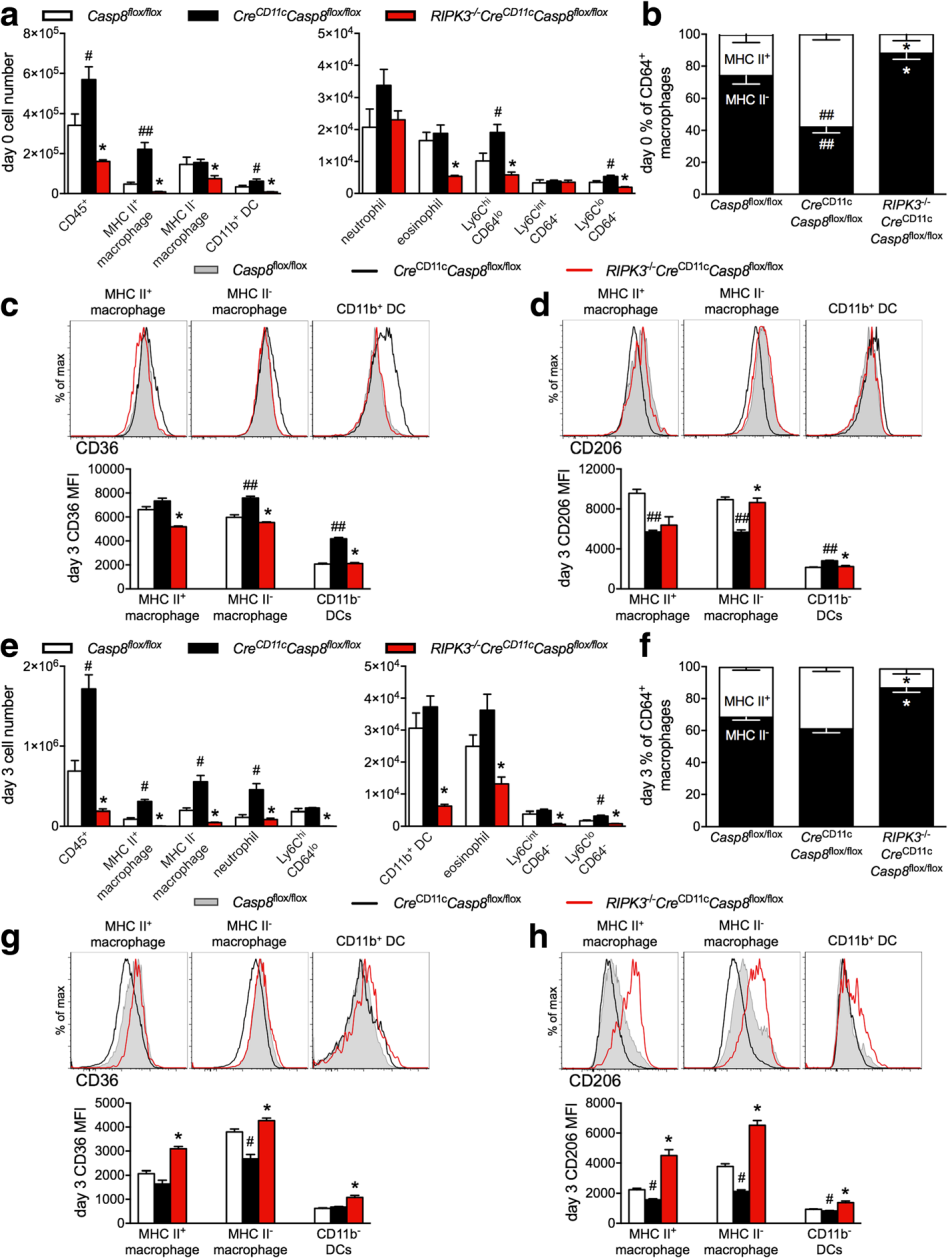
Receptor-interacting serine-threonine kinase 3 (RIPK3) deletion reverses the dyregulation of the joint induced by caspase-8 deficiency under steady state and arthritic conditions. **a**-**d** Ankles from naïve 10–12-week-old male *Casp8* ^flox/flox^ (control, n = 5), *Cre* ^CD11c^ *Casp8* ^flox/flox^ (n = 5) and *RIPK3* ^–/–^ *Cre* ^CD11c^ *Casp8* ^flox/flox^ (n = 3) mice were analyzed by flow cytometric analysis. **a** Day-0 synovial cell distribution presented as numbers of cells. **b** Day-0 proportion of macrophages (CD11b^+^CD64^+^) that are major histocompatibility complex (MHC) II^+^ and MHC II^–^. **c** Day-0 CD36 expression on MHC II^+^ macrophages (CD11b^+^CD64^+^MHCII^+^), MHC II^–^ macrophages (CD11b^+^CD64^+^MHCII^-^) and CD11b^+^ dendritic cells (DCs) (CD11c^+^CD11b^+^CD64^-^MHCII^+^). **d** Day-0 CD206 expression on MHC II^+^ macrophages, MHC II^–^ macrophages and CD11b^+^ DCs. **e**-**h** Ankles from 10–12-week-old male control (n = 4), *Cre* ^CD11c^ *Casp8* ^flox/flox^ (n = 4) and *RIPK3* ^–/–^ *Cre* ^CD11c^ *Casp8* ^flox/flox^ (n = 4) mice on day 3 post induction of K/BxN serum-transfer-induced arthritis were analyzed by flow cytometric analysis. **e** Day-3 synovial cell distribution represented as numbers of cells. **f** Day-3 proportion of macrophages that are MHC II^+^ and MHC II^–^. **g** Day-3 CD36 expression on MHC II^+^ macrophages, MHC II^–^ macrophages and CD11b^+^ DCs. **h** Day-3 CD206 expression on MHC II^+^ macrophages, MHC II^–^ macrophages and CD11b^+^ DCs. Data are means ± SEM, representative of two individual studies and are compared between control and *Cre* ^CD11c^ *Casp8* ^flox/flox^ mice by the Mann-Whitney test: ^#^ *p* < 0.05; ^##^ *p* < 0.005 and between *Cre* ^CD11c^ *Casp8* ^flox/flox^ and *RIPK3* ^–/–^ *Cre* ^CD11c^ *Casp8* ^flox/flox^ mice by the Mann-Whitney test: *p < 0.05

Since caspase-8 may mediate its suppressive effect during K/BxN serum-transfer-induced arthritis independent of preventing RIPK3-mediated necroptosis, we sought to determine alternate modalities by which this RIPK3-mediated suppression potentially occurs. We therefore interrogated naïve and arthritic joints from *Casp8* ^flox/flox^, *Cre* ^CD11c^ *Casp8* ^flox/floxl^ and *RIPK3* ^–/–^ *Cre* ^CD11c^ *Casp8* ^flox/flox^ mice by flow cytometric analysis. We detected increased numbers of CD45^+^ hematopoeitic cells in the joint of *Cre* ^CD11c^ *Casp8* ^flox/flox^ mice at steady state compared to control *Casp8* ^flox/flox^ joints owing to elevated numbers of MHC II^+^ macrophages, CD11b^+^ DCs, Ly6C^hi^CD64^lo^ cells and Ly6C^lo^CD64^-^ cells (Fig. 5a). Further, *Cre* ^CD11c^ *Casp8* ^flox/flox^ joints exhibited an increased proportion of MHC II^+^ macrophages and a correspondingly reduced proportion of MHC II^–^ tissue-resident macrophages (Fig. 5b). We then examined expression levels of CD36, a scavenger receptor that participates in the internalization of apoptotic cells and modified low-density lipoproteins, and CD206 (C-type mannose receptor 1), a protein active in endocytosis/phagocytosis, within the macrophage and DC populations of the naïve joint. *Cre* ^CD11c^ *Casp8* ^flox/flox^ MHC II^+^ and MHC II^–^ macrophage populations and DCs expressed elevated levels of CD36, compared to control *Casp8* ^flox/flox^ populations (Fig. 5c). Further, *Cre* ^CD11c^ *Casp8* ^flox/flox^ MHC II^+^ and MHC II^–^ macrophage populations presented with reduced levels of CD206, compared to control *Casp8* ^flox/flox^ populations, while *Cre* ^CD11c^ *Casp8* ^flox/flox^ CD11b^+^ DCs displayed elevated expression CD206 compared to *Casp8* ^flox/flox^ CD11b^+^ DCs (Fig. 5d). Strikingly, deletion of RIPK3 prevented these cellular alterations in caspase-8 deficient naïve joints (Fig. 5a-d).

Next we assessed the arthritic joint at day 3, in which the initiation of inflammation was greater in *Cre* ^CD11c^ *Casp8* ^flox/flox^ mice than control *Casp8* ^flox/flox^ mice as determined from Fig. 3e. *Cre* ^CD11c^ *Casp8* ^flox/flox^ mice continued to show increased CD45^+^ hematopoeitic cells in the joint at day 3 due to elevated numbers of MHC II^+^ and MHC II^–^ macrophages, neutrophils and Ly6C^lo^CD64^-^ cells (Fig. 5e). However, the disparity in proportion of MHC II^+^ and MHC II^–^ macrophages in *Cre* ^CD11c^ *Casp8* ^flox/flox^ joints at steady state was no longer apparent at day 3 following K/BxN serum-transfer-induced arthritis induction (Fig. 5f). Further, MHC II^+^ and MHC II^–^ macrophage populations showed a reduction in both CD36 and CD206 expression compared to control *Casp8* ^flox/flox^ populations, while no difference was observed in the expression of these markers between experimental and control CD11b^+^ DC populations (Fig. 5g, h). Similar to naïve joints, deletion of RIPK3 in *Cre* ^CD11c^ *Casp8* ^flox/flox^ mice prevented the aberrant presence of increased hematopoietic cells observed in *Cre* ^CD11c^ *Casp8* ^flox/flox^ joints (Fig. 5e, f), and restored expression of CD36 and CD206 to levels similar to control *Casp8* ^flox/flox^ populations (Fig. 5g, h). Taken together, these data provide evidence that implicates the caspase-8-RIPK3 signaling axis in the maintenance of synovial populations under both steady state and inflammatory conditions that are capable of either driving or preventing K/BxN serum-transfer-induced arthritis.

## Discussion

Rheumatoid arthritis (RA) affects nearly 1% of the world’s population, making it one of the most prevalent autoimmune diseases. While aberrant monocyte/macrophage and DC function have been detected in the RA synovium, the underlying mechanisms remain largely a mystery. We show here that caspase-8 in lysozyme M-expressing cells promotes a prolonged inflammatory response, as *Cre* ^CD11c^ *Casp8* ^flox/flox^ mice exhibit reduced severity and accelerated resolution of K/BxN serum-transfer-induced arthritis. In contrast, loss of caspase-8 in CD11c-expressing cells controls the magnitude of the initial inflammatory response, as *Cre* ^CD11c^ *Casp8* ^flox/flox^ mice have accelerated induction and exacerbated severity of the effector phase of disease. These data suggest that intact caspase-8 signaling maintains opposing roles in lysozyme M-expressing and CD11c-expressing cells in the pathogenesis of RA. Interestingly, in both cre recombinase constructs, caspase-8 is deleted in both of the synovial macrophage subpopulations and the CD11b^+^ DCs in the naïve joint. However, in the spleen, a secondary lymphoid organ, caspase-8 is restricted to the neutrophil and monocyte/macrophage populations in *Cre* ^LysM^ *Casp8* ^flox/flox^ mice and the conventional DC populations in *Cre* ^CD11c^ *Casp8* ^flox/flox^ mice. We postulate that caspase-8 deletion in the neutrophils and monocytes of *Cre* ^LysM^ *Casp8* ^flox/flox^ mice that enter the joint under inflammatory conditions may contribute to the differing responses to K/BxN serum-transfer-induced arthritis in *Cre* ^LysM^ *Casp8* ^flox/flox^ and *Cre* ^CD11c^ *Casp8* ^flox/flox^ mice. These data suggest that the specificity of the *Cre* ^LysM^ and *Cre* ^CD11c^ deletion constructs are not so clear within tissue-resident populations.

We previously demonstrated that circulating Ly6C^lo^ monocytes are critical for the induction of K/BxN serum-transfer-induced arthritis. Further, we showed that naïve murine joints contain both MHC II^+^ and MHC II^–^ macrophages, with the majority being MHC II^–^ tissue-resident macrophages that are capable of limiting the initiation of K/BxN serum-transfer-induced arthritis [50]. Here we show that *Cre* ^CD11c^ *Casp8* ^flox/flox^ mice possess an increased population of Ly6C^lo^ monocytes, which potentially facilitate the observed accelerated initiation of K/BxN serum-transfer-induced arthritis. *Cre* ^CD11c^ *Casp8* ^flox/flox^ mice are also predisposed to a reduced proportion of MHC II^–^ macrophages in the naïve joint, suggesting that the lack of a sufficient population of these cells at the onset of disease may contribute to the accelerated initiation of arthritis. Further, we show that caspase-8 potentially controls the endocytic capacity of macrophages, as caspase-8-deficient synovial macrophages in *Cre* ^CD11c^ *Casp8* ^flox/flox^ mice express reduced CD206. Although the M1/M2 macrophage classification system may not be entirely relevant beyond in vitro settings, elevated CD206 expression has been associated with M2 alternatively activated macrophages that participate in wound healing and remission and/or prevention of disease [54]. It is possible that the observed reduction in CD206 may render caspase-8-deficient macrophages less capable of endocytosing cellular debris arising from the damage induced by the arthritic inflammatory assault, and are therefore unable to control the ensuing inflammation. Further, based on in vitro studies, M2 macrophages exhibit poor antigen-presentation capabilities unlike classically activated macrophages [54]. This reduction of CD206 may be indicative of a smaller proportion of M2-like macrophages in the joints of *Cre* ^CD11c^ *Casp8* ^flox/flox^ mice. Therefore, caspase-8-deficient synovial macrophages may show increased antigen-presenting capabilities. Thus, further analysis is required to determine how reduced CD206 affects synovial macrophage function. Taken together, these data suggest that within the joint, the caspase-8/RIPK3 signaling axis controls macrophage function, potentially not through death-related mechanisms.

The function of caspase-8 extends to inhibition of signaling through RIPK, a family of enzymes that turn on programmed necrotic cell death, or necroptosis. However, RIPK inhibition by caspase-8 not only leads to suppression of necroptosis but also potentially death-independent, cell-specific processes including inflammation. We have previously shown that caspase-8 controls the response to TLR activation in monocytes/macrophages, while caspase-8 limits DC activation and prevents a break in tolerance, and both of these functions are RIPK-dependent. In vitro studies implicate RIPK1 in the hyper-activation of bone marrow-derived macrophages and bone marrow-derived DCs from *Cre* ^LysM^ *Casp8* ^flox/flox^ and *Cre* ^CD11c^ *Casp8* ^flox/flox^ mice following TLR activation [37, 38]. These data suggest that uncontrolled RIPK1 activity might contribute to the systemic lupus erythematosus (SLE)-like symptoms of caspase-8-deficient mice. Further, symptoms of systemic inflammation in myeloid cell-specific caspase-8-deficient mice are ameliorated by deletion of RIPK3 [37]. However, we find that RIPK3 is not involved in the aggressive SLE-like disease in *Cre* ^CD11c^ *Casp8* ^flox/flox^ mice [38, 55]. While a recent report shows that *RIPK3* ^–/–^ mice display more rapid resolution of K/BxN serum-transfer-induced arthritis compared to control mice [56], we did not observe this pattern in our study, which may be the result of K/BxN serum differences, colony environment and/or diet. We did find that *RIPK3* ^–/–^ *Casp8* ^flox/flox^ mice have slower induction of arthritis than *RIPK3* ^–/–^ mice, potentially owing to interactions from the remaining 129 background present in both *Casp8* ^flox/flox^ and *RIPK3* ^–/–^ strains despite being backcrossed to B6 for over 12 and 4 generations, respectively [26, 37, 38, 46]. However, we observed that global deletion of RIPK3 in *Cre* ^LysM^ *Casp8* ^flox/flox^ and *Cre* ^CD11c^ *Casp8* ^flox/flox^ mice causes the response to K/BxN serum-transfer-induced arthritis to revert to that of *Casp8* ^flox/flox^ control mice, indicating that the aberrant responses observed require RIPK3 action. Further, global deletion of RIPK3 in *Cre* ^CD11c^ *Casp8* ^flox/flox^ mice reversed the response to levels below that of *Casp8* ^flox/flox^ mice, potentially indicating a caspase-8-independent effect of RIPK3 in *Cre* ^CD11c^ *Casp8* ^flox/flox^ mice; future studies are required to understand this phenomenon. Caspase-8 initiates the degradative phase of the apoptotic cascade. However, in our mixed bone marrow chimera mice, *Cre* ^CD11c^ *Casp8* ^flox/flox^-derived synovial macrophage subsets were detected at decreased proportions compared to WT-derived subsets, indicating that caspase-8-deficient synovial macrophage subsets do not accumulate and/or persist in the joint due to a lack of apoptosis. One interpretation of these data is that synovial macrophage subsets are succumbing to RIPK3-mediated necroptosis, since caspase-8 is not present to inhibit RIPK3. However, the proportion of *RIPK3* ^–/–^ *Cre* ^CD11c^ *Casp8* ^flox/flox^-derived macrophage subsets were not restored to WT proportions, indicating that caspase-8-deficient macrophages are not undergoing necroptosis in the naïve joint. These data point to a death-independent function for the caspase-8/RIPK3 signaling axis within the joint; however, further investigation will be required to determine potential mechanisms.

IL-1β is a key inflammatory cytokine in the pathogenesis of RA, as highlighted by its role in driving cartilage destruction and the efficacy of its blockade in both mice and humans [57]. IL-1β is predominantly produced by innate immune cells through activation of the NLRP3 inflammasome [58]. Humanized mice expressing disease-associated mutations in NLRP3 develop normally but acquire progressive and debilitating arthritis with age [59]. Numerous studies have implicated caspase-8 in controlling activation of the NLRP3 inflammasome. Loss of caspase-8 in DCs and macrophages facilitates LPS-induced and Pam_3_Cys-induced NLRP3 activation through RIPK3 [55, 56, 60]. Further, in the absence of both inhibitors of apoptosis (IAPs) and caspase-8, RIPK3-mediated NLRP3 inflammasome activation can occur [56]. However, in contrast, under certain circumstances, caspase-8 can directly cleave pro-IL-1β into its mature form [61–63], suggesting that caspase-8 is necessary for IL-1β production. Indeed, a recent study suggests that whole blood cells from patients with RA show increased expression of NLRP3 and secretion of NLRP3-mediated IL-1β via TLR3 and TLR4, but not TLR2, activation that is driven by caspase-1 and caspase-8 [64]. While we do not see differences in serum IL-1β between our strains, we have not yet examined IL-1β within the joint. Taken together, depending on the stimulus, the delicate balance of caspase-8/RIPK3 signaling axis may be controlling NLRP3 inflammasome activation within the arthritic joint and warrants further investigation.

A recent study examined an SNP within *Caspase*-*8* in the context of a large Chinese-based cohort (615 patients with RA and 839 controls) and found no association between this particular SNP and susceptibility to RA development [65]. However, a prior genome-wide association study identified an SNP associated with risk of RA development within the locus containing the gene encoding for both caspase-8 and the catalytically inactive homolog of caspase-8, cFLIP [39]. It has been shown that mice with targeted deletion of cFLIP in CD11c-expressing populations develop spontaneous erosive inflammatory arthritis that resembles RA and is accompanied by the production of autoantibodies to joint antigens [66]. However, a report by a different research group shows that mice lacking cFLIP in CD11c-expressing cells were found to develop neutrophilia (caused by excessive production of granulocyte colony-stimulating factor receptor, (G-CSF)) and splenomegaly, but do not spontaneously develop arthritis, potentially owing to either differences in the efficiency of cFLIP deletion or variability in colony environment [67–70]. In contrast to both models of CD11c-specific deletion of cFLIP, caspase-8 deletion in CD11c-expressing populations does not result in the spontaneous development of arthritis or neutrophilia. This suggests that although cFLIP is a catalytically inactive form of caspase-8, these molecules possess differing functions within CD11c-expressing populations, and further examination is warranted to determine if the RA-risk SNP affects cFLIP or caspase-8.

Caspase-8 is a downstream signaling mediator of the death receptor Fas, which has been implicated in inducible murine models of RA-like disease [71, 72]. The onset of K/BxN serum-transfer-induced arthritis in lysozyme-M-specific *Fas*-knockout (*Cre* ^LysM^ *Fas* ^flox/flox^) mice is comparable to that of control *Fas* ^flox/flox^ mice. However, arthritis resolution is accelerated in the chronic phase in *Cre* ^LysM^ *Fas* ^flox/flox^ mice, as evidenced by the reduction of inflammation and neutrophil infiltration [73]. Consistent with this rapid resolution of disease, higher levels of IL-10 and reduced CXCL5 (a neutrophil chemotactic chemokine) and TLR2 ligand, endoplasmin (also known as GRP94) [73], are expressed in the joints of *Cre* ^LysM^ *Fas* ^flox/flox^ mice. Here we show that *Cre* ^LysM^ *Casp8* ^flox/flox^, similar to *Cre* ^LysM^ *Fas* ^flox/flox^ mice, exhibit accelerated resolution of K/BxN serum-transfer-induced arthritis. This finding suggests that in lysozyme-expressing cells, Fas and caspase-8 function may be involved in the same pathway to control IL-10 expression to enable sufficient response to an inflammatory insult.

We believe that future studies will elucidate new cell autonomous mechanisms by which caspase-8 regulates arthritis pathogenesis. Here, we provide a potential mechanism between the link between caspase-8 and RA susceptibility and the cellular mechanisms by which this predisposition takes effect that includes suppression of inflammation induced by RIPK3. Therefore, these studies substantiate critical and opposing cell-specific roles for the caspase-8/RIPK3 signaling axis in arthritis pathogenesis and highlight the need for further mechanistic insight.

## Conclusions

Since patients with RA often fail to achieve remission using current immunosuppressive and biologic therapies and side effects from treatment are substantial, the ultimate objective is to utilize these research discoveries that link established susceptibility to RA development with caspase-8/RIPK3 signaling in macrophages and DCs to assist in the development of safer and more effective therapies.

## Additional file

Additional file 1: Table S1. List of antibodies utilized for circulating and synovial cell flow cytometric analysis. **Figure S1.** Serum cytokine levels in *Casp8* ^flox/flox^, *Cre* ^LysM^ *Casp8* ^flox/flox^ and *Cre* ^CD11c^ *Casp8* ^flox/flox^ mice before and during K/BxN serum-transfer-induced arthritis. **Figure S2.** K/BxN serum transfer-induced arthritis in control strains. **Figure S3.** Caspase-8 deletion in synovial macrophages and dendritic cells of the naïve joint. **Figure S4.** Gating strategy for synovial population distribution in mixed bone marrow chimeric joints. (DOCX 463 kb)
