# Supplementary material for: The caspase-8/RIPK3 signaling axis in antigen presenting cells controls the inflammatory arthritic response
Source: Arthritis Res Ther. 2017 Oct 4;19:224. doi: 10.1186/s13075-017-1436-4 (PMC5628498; doi:10.1186/s13075-017-1436-4)
Supplement: Additional file 1: Table S1. — List of antibodies utilized for circulating and synovial cell flow cytometric analysis. Figure S1. Serum cytokine levels in Casp8 flox/flox, Cre LysM Casp8 flox/flox and Cre CD11c Casp8 flox/flox mice before and during K/BxN serum-transfer-induced arthritis. Figure S2. K/BxN serum transfer-induced arthritis in control strains. Figure S3. Caspase-8 deletion in synovial macrophages and dendritic cells of the naïve joint. Figure S4. Gating strategy for synovial population distribution in mixed bone marrow chimeric joints. (DOCX 463 kb) [file 13075_2017_1436_MOESM1_ESM.docx]

**The caspase-8/RIPK3 signaling axis in antigen presenting cells controls the inflammatory arthritic response**

Salina Dominguez, BS, Anna B. Montgomery, DPhil, G. Kenneth Haines III, MD, Christina L. Bloomfield, BS and Carla M. Cuda, PhD

**Additional file 1**

**Table S1.** List of antibodies utilized for circulating and synovial cell flow cytometric analysis.

| **Antigen** | **Clone** | **Fluorochrome** | **Manufacturer** |
| --- | --- | --- | --- |
| CD45 | 30-F11 | FITC | eBioscience |
| CD45 | 30-F11 | V500 | BD Biosciences |
| CD45.1 | A20 | PerCP-Cy5.5 | eBioscience |
| CD45.2 | 104 | FITC | BD Biosciences |
| CD11b | M1/70 | PerCP-Cy5.5 | BD Biosciences |
| CD11b | M1/70 | efluor 450 | eBioscience |
| CD11b | M1/70 | Alexa 700 | BD Biosciences |
| MHC II | M5/114.15.2 | efluor 450 | eBioscience |
| CD206 | MR5D3 | Alexa 647 | AbD Serotec |
| CD36 | CRF D-2712 | APC | BD Biosciences |
| Ly6C | AL-21 | APC-Cy7 | BD Biosciences |
| CD64 | X54-5/7.1 | PE | Biolegend |
| Siglec F | E50-2440 | PE-CF594 | BD Biosciences |
| Ly6G | 1A8 | PE-Cy7 | Biolegend |
| Ly6G | 1A8 | PerCP-Cy5.5 | BD Biosciences |
| CD115 | AFS98 | PE | eBioscience |
| B220 | RA3-6B2 | PE-CF594 | BD Biosciences |
| NK1.1 | PK136 | Alexa700 | BD Biosciences |
| CD62L | MEL-14 | PE-Cy7 | eBioscience |

**Figure S1. Serum cytokine levels in *Casp8*^flox/flox^, *Cre*^LysM^*Casp8*^flox/flox^ and *Cre*^CD11c^*Casp8*^flox/flox^ mice before and during K/BxN serum transfer-induced arthritis.** 10-12 week old male *Casp8*^flox/flox^ (control, n=5), *Cre*^LysM^*Casp8*^flox/flox^ (n=5) and *Cre*^CD11c^*Casp8*^flox/flox^ (n=5) mice were intravenously injected with K/BxN serum. Depicted day 0 and 7 serum cytokine levels are representative of two individual studies. Differences between control and *Cre*^LysM^*Casp8*^flox/flox^ or *Cre*^CD11c^*Casp8*^flox/flox^ mice are compared by 2-way ANOVA with Bonferroni post-test.

**Figure S2. K/BxN serum transfer-induced arthritis in control strains.** 10-12 week old male B6 (n=10), *RIPK3*^–/–^ (n=12), *Casp8*^flox/flox^ (n=14) and *RIPK3*^–/–^*Casp8*^flox/flox^ (n=4) mice were intravenously injected with K/BxN serum. Depicted are combined ‘change in ankle width’ and ‘clinical score’ from two individual experiments. Differences between *Casp8*^flox/flox^ and other groups compared by 2-way ANOVA with Bonferroni post-test.

**Figure S3. Caspase-8 deletion in synovial macrophages and dendritic cells of the naïve joint.** Synovial MHC II^+^ macrophages, MHC II^-^ macrophages and CD11b^+^ dendritic cells were sorted from ankles of naïve 10-12 week old male *Casp8* ^flox/flox^ (control), *Cre*^LysM^*Casp8*^flox/flox^ and *Cre*^CD11c^*Casp8*^flox/flox^ mice and DNA was analyzed for the presence of the caspase-8 floxed allele. Data are presented as % deletion and are derived as follows: divide the level of the floxed allele from a *Cre*^LysM^*Casp8*^flox/flox^ - and *Cre*^CD11c^*Casp8*^flox/flox^ -sorted population by the level of the floxed allele from the *Casp8*^flox/flox^ -sorted population, convert the resulting value into % and then subtract from 100.

**Figure S4. Gating strategy for synovial population distribution in mixed bone marrow chimeric joints.** FACS plots from a mixed bone marrow chimera joint. Red arrows denote sequential gated population (red boxes). Black arrows denote sequential non-gated population.
